# Algebraic differentiation for fast sensitivity analysis of optimal flux modes in metabolic models

**DOI:** 10.1093/bioinformatics/btaf287

**Published:** 2025-05-06

**Authors:** Hester Chapman, Miroslav Kratochvíl, Oliver Ebenhöh, St Elmo Wilken

**Affiliations:** Institute of Quantitative and Theoretical Biology, Heinrich Heine University, Düsseldorf 40255, Germany; Luxembourg Centre of Systems Biomedicine, University of Luxembourg, Esch-sur-Alzette L-4367, Luxembourg; Institute of Quantitative and Theoretical Biology, Heinrich Heine University, Düsseldorf 40255, Germany; Institute of Quantitative and Theoretical Biology, Heinrich Heine University, Düsseldorf 40255, Germany

## Abstract

**Motivation:**

Sensitivity analysis is a useful tool to identify key parameters in metabolic models. It is typically only applied to the growth rate, disregarding the sensitivity of other solution variables to parameters. Further, sensitivity analysis of elementary flux modes could provide low-dimensional insights into optimal solutions, but they are not defined when a model is subject to inhomogeneous flux constraints, such as the frequently used ATP maintenance reaction.

**Results:**

We introduce optimal flux modes (OFMs), an analogue to elementary flux modes (EFMs), but specifically applied to optimal solutions of constraint-based models. Further, we prove that implicit differentiation can always be used to efficiently calculate the sensitivities of both whole-model solutions and OFM-based solutions to model parameters. This allows for fine-grained sensitivity analysis of the optimal solution, and investigation of how these parameters exert control on the optimal composition of OFMs. This novel framework is implemented in DifferentiableMetabolism.jl, a software package designed to efficiently differentiate solutions of constraint-based models. To demonstrate scalability, we differentiate solutions of 342 yeast models; additionally we show that sensitivities of specific subsystems can guide metabolic engineering. Applying our scheme to an *Escherichia coli* model, we find that OFM sensitivities predict the effect of knockout experiments on waste product accumulation. Sensitivity analysis of OFMs also provides key insights into metabolic changes resulting from parameter perturbations.

**Availability and implementation:**

Software introduced here is available as open-source Julia packages DifferentiableMetabolism.jl (https://github.com/stelmo/DifferentiableMetabolism.jl) and ElementaryFluxModes.jl (https://github.com/HettieC/ElementaryFluxModes.jl), which both work on all major operating systems and computer architectures. Code to reproduce all results is available from https://github.com/HettieC/DifferentiableOFMPaper, and as an archive from https://doi.org/10.5281/zenodo.15183208.

## 1 Introduction

Genome-scale metabolic models (GSMMs) provide a framework for studying cellular metabolism (Varma and Palsson 1994, Edwards and Palsson 1999). The annotated genome of an organism is used to identify metabolic reactions with an associated catalysing enzyme. In the simplest case, GSMMs are simulated using flux balance analysis (FBA) (Orth *et al.* 2010). Main applications include metabolic engineering (Park *et al.* 2009), investigating human metabolism and disease (Brunk *et al.* 2018), and pathogen–drug target identification (Kim *et al.* 2010).

While FBA simulations often capture the function of essential genes, they are less successful in predicting quantitative changes in phenotypic behaviour. The seemingly wasteful strategies of overflow metabolism in bacteria, and the Crabtree effect in yeast, cannot be reproduced through FBA without imposing ad-hoc flux constraints on selected reactions. It has been posited that overflow metabolism in *Escherichia coli* results from constrained protein allocation in the cell (Basan *et al.* 2015, Bruggeman *et al.* 2020); to account for this phenomenon in GSMMs, total proteome constraints and enzyme kinetics can be added to create an enzyme-constrained genome-scale metabolic model (ecGSMM) (Beg *et al.* 2007, Sánchez *et al.* 2017).

Parameterization of an ecGSMM requires knowledge of both the molar mass and turnover number ($k_{\text{cat}}$) of each enzyme. While the molar masses of enzymes are typically available from databases, the $k_{\text{cat}}$-values are poorly characterized (Davidi and Milo 2017). Due to this lack of reliable experimental data, researchers may have to turn to non-mechanistic approximations via machine learning, for example that of Kroll *et al.* (2023).

Despite these parameterization issues, ecGSMMs are still able to effectively capture a much wider variety of phenotypes than GSMM. It is thus of interest to analyse which parameters exert the most control over an optimal flux distribution, as this can be used to guide metabolic engineering efforts or uncover novel drug targets. Previous work has detailed how to implicitly differentiate an optimal solution to an ecGSMM to find the sensitivity of a solution to parameter changes (Wilken *et al.* 2022). However, well-defined criteria for when this is possible were not provided.

Alternatively, sensitivity analysis of ecGSMMs can also be performed through finite differentiation, where a parameter is perturbed and the change in the values of the model variables is used to approximate the sensitivity of those variables to the perturbed parameter. The main drawback is inefficiency, with *n* parameters requiring at least *n* additional linear programs to be solved, depending on the finite differencing scheme. Furthermore, numerical errors can arise, with accuracy dependent on the size of the perturbation. Another method, based on the shadow prices of linear optimization problems, has also been extensively studied (Liebermeister 2018, van den Bogaard *et al.* 2024). A major drawback of the shadow price approach is the inability to calculate sensitivities of variables other than the objective function.

Here, we prove that it is generally possible to implicitly differentiate a flux solution of an ecGSMM, and show that the only criterion necessary for this is the removal of inactive reactions from the model before differentiation. Moreover, when the inactive reactions are removed, we show that the solution is unique and the derivatives are well-defined.

Further, one can always decompose an optimal solution of an ecGSMM with homogeneous constraints into a linear combination of a small number of elementary flux modes (EFMs) (De Groot *et al.* 2019). However, when inhomogeneous constraints are included, such as an ATP maintenance reaction, EFMs are not defined. We introduce optimal flux modes (OFMs), an analogue to EFMs, which resolve the inhomogeneity issue. We show that OFMs provide a similar low-dimensional view of metabolic pathways as EFMs, but restricted to modes fulfilling the optimal objective value.

To demonstrate the utility of this approach, we investigate the OFMs associated with an enzyme-constrained model exhibiting overflow metabolism. We find both a respiratory and a fermentative OFM in the optimal solution. It may be of interest to find out to what extent the enzyme kinetic parameters control the phenotypic ratio of respiration and fermentation; for example, how would a knock down of a glycolytic enzyme affect the optimal proportion of the two OFMs? In this work, we prove that the sensitivity of OFM usage to model parameters can be calculated using the same implicit algebraic differentiation technique.

To enable efficient differentiation of constraint-based models, we introduce DifferentiableMetabolism.jl, a Julia package for the fast calculation of sensitivities of all model variables to parameters. The framework uses implicit differentiation, analogous to classic metabolic control analysis (MCA) (Kacser and Burns 1973). We also present ElementaryFluxModes.jl to compute and implicitly differentiate EFMs or OFMs. We use these packages to differentiate the optimal solutions of 342 previously published fungal models (Li *et al.* 2022), showing that the method is readily applicable on a large-scale. Finally, our investigation into the minimization of acetate production in *E. coli* highlights one possible application of our framework in metabolic engineering.

## 2 Theory

Here, we describe the ecGSMM model formulation, introduce and define optimal flux modes, and summarize the established method of implicit differentiation to find sensitivities of optimal solutions (Wilken *et al.* 2022). Detailed proofs of all lemmas and theorems are presented in the Supplementary Data.

### 2.1 Model formulation

Following the model formulation from Sanchez (Sánchez *et al.* 2017), we describe the enzyme-constrained FBA (ecFBA) optimization problem applied to a simple ecGSMM as follows:

(1) $$\begin{array}{cc} \text{maximise} & v_{r}\\ \text{subject}\;\text{to} & (C1)\;\;\mathrm{Sv}=0\\ & (C2)\;\;v_{i}\geq 0\\ & (C3)\;\;v_{i}=k_{\text{cat},i}\cdot e_{i}\;\;\forall i\in R\\ & \left(C4)\;\;\sum_{i\in R_{k}}e_{i}\leq E_{k}\;\;\forall k\in [1,\ldots ,K\right] \end{array}$$

There are *r* reactions in the model, and we reorder them such that $v_{r}$ is the objective reaction. The set $R\subset N^{n}$ is the set of indices of reactions with associated $k_{\text{cat}}$-values. The flux, $v_{i}$, through reaction *i* is determined by the concentration, $e_{i}$, of the enzyme catalysing the reaction, multiplied by its associated turnover number, $k_{\text{cat},i}$. The number of enzyme pools is given by *K*, and $R_{k}$ gives the set of indices of enzymes in the *k*-th pool. Reversible reactions are split into a forward and backward irreversible reaction. Enzyme complexes and isozymes may be included in the model formulation, but for brevity are not included in equation (1). We constrain the proteome capacity of the model, with the total usage of enzymes from the *k*-th pool bounded by the capacity $E_{k}$.

In the above formulation, one places bounds $E_{k}$ on a subset of model enzymes in a particular pool *k*. This pool could be a compartment, say the membrane, a specific pathway, or an individual enzyme. The stoichiometric matrix, $S\in R^{m\times r}$, contains a row for each of the *m* metabolites and a column for each of the *r* reactions, with entries in each column giving the stoichiometric coefficients of the metabolites involved in that column’s reaction (Orth *et al.* 2010). This is the matrix we use to find elementary flux modes of a network.

### 2.2 Flux cones and elementary flux modes

The set of flux vectors v satisfying both Sv=0 and $v_{i}\geq 0$ forms a convex polyhedral cone, called the flux cone (Klamt *et al.* 2017):

(2) $$\text{FC}(S)=\{v\in R^{n}|\mathrm{Sv}=0,v_{i}\geq 0\}$$

Any non-trivial flux vector v is called an admissible flux mode if it is contained in the flux cone FC(S)  (2). We define the support of a mode, $\mathrm{supp}(v)=\{i|v_{i}\neq 0\}$ as the set of indices of non-zero elements in the flux mode v.

Definition 1 (Elementary flux mode (EFM)). *An admissible flux mode* v∈FC(S)  *is called an elementary flux mode*, m*, if there exists no other admissible flux mode* v*, where* supp(m)⊃supp(v)*. Thus*, supp(m)  *cannot be written as a proper superset of any other mode* v.

From this definition, if a flux mode is elementary, it has minimal support, and any attempt to reduce its support by deleting a reaction will imply v=0.

### 2.3 Inhomogeneous constraints and optimal flux modes

In genome-scale metabolic modelling, there are often not only homogeneous (v≥0) but also inhomogeneous constraints (v≥c>0 for some constant *c*). For example, uptake and secretion rates of metabolites may be constrained from exometabolomics data, ${}^{13}C$-metabolic flux analysis allows for quantification of intracellular fluxes, and proteomics or transcriptomics may be used to constrain enzyme concentrations. It is important that we analyse these types of model formulations. Incorporating inhomogeneous equality constraints into (1) can be done as follows:

(3) $$\begin{array}{cc} \text{maximise} & v_{r}\\ \text{subject}\;\text{to} & \left(C1-C4\right)\\ & (C5)\;\;v_{j}\geq c_{j}>0\;\forall j\in R_{f} \end{array}$$

where $R_{f}$ is the set of reactions with fixed non-zero flux, and $c_{j}$ is the known constant flux of reaction *j*.

The solution space of a model with the setup given by problem (3) will no longer form a flux cone, but rather the flux polyhedron P(S), defined as:

(4) $$P(S)=\{v\in R^{n}|\mathrm{Sv}=0,\;v_{i}\geq 0,\;v_{j}\geq c_{j}\;\forall j\in R_{f}\}$$

We distinguish between the homogeneous problem (1) and the inhomogeneous problem (3) since EFMs are not defined for flux polyhedra such as (4) (Klamt *et al.* 2017). We can, however, incorporate inhomogeneous equality constraints into a modified stoichiometric matrix. Any inhomogeneous constraints that are active (fulfilled with equality) in an optimal solution may be treated as equality constraints, and we treat inhomogeneous constraints that are inactive as homogeneous constraints.

Following the approach of Schuster and Schuster (1993), we may introduce a slack variable $\bar{v}$ to account for inhomogeneous constraints. Let $v^{\left(1\right)}$ and $S^{\left(1\right)}$ contain the flux rates and stoichiometries of reactions with unknown fluxes, and let $w:=S^{\left(2\right)}v^{\left(2\right)}$ contain those of the reactions with fixed known fluxes. We may now write an optimization problem involving only homogeneous constraints:

(5) $$\begin{array}{cc} \text{maximise} & \bar{v}\\ \text{subject}\;\text{to} & \mathrm{Ax}=0\\ & x_{i}\geq 0\\ & x_{i}=k_{\text{cat},i}\cdot e_{i}\;\;\forall i\in R\\ & \sum_{i\;\in \;R_{k}}e_{i}\leq E_{k}\;\;\forall k\in [1,\ldots ,K]\\ \text{where} & A:=\left[\begin{array}{cc} S^{\left(1\right)} & w \end{array}\right],\;x:=\left[\begin{array}{c} v^{\left(1\right)}\\ \bar{v} \end{array}\right]. \end{array}$$

When $\bar{v}=1$, solutions to the homogeneous system (5) coincide precisely with solutions of problem (3).

A non-trivial vector x is called an admissible flux mode of (3) if it is contained in the optimal flux cone

(6) $$\text{OFC}(A)=\{x\in R^{n}|\mathrm{Ax}=0,v_{i}\geq 0\}$$

Definition 2 (Optimal flux mode (OFM)). *A flux mode* $x=\left[\begin{array}{c} v^{\left(1\right)}\\ \bar{v} \end{array}\right]$  *is called an optimal flux mode of*  (3)  *if it fulfils the following conditions:*
 

- *Admissibility:* x∈OFC(A)
- *Minimal support: There exists no other flux mode* y  *satisfying the above conditions, where*  supp(x)⊃supp(y)
- *Optimality:* $\bar{v}=1$.

The first two conditions for an OFM coincide with Definition 1 of an EFM of the optimal flux cone (6); it is the extra condition of optimality that distinguishes OFMs from EFMs. From Definition 2, an OFM is an optimal, minimal, flux-carrying mode. Should a reaction be deleted from an OFM, either the mode would no longer be able to carry flux, or it would do so at a sub-optimal rate. A mode with a sub-optimal flux is one in which the ratio of $v_{r}$ to fixed fluxes $v^{\left(2\right)}$ is lower than that in the optimal solution. A similar construction, elementary flux vectors, has previously been reported (Urbanczik 2007, Klamt *et al.* 2017), but while OFMs are support minimal, the set of elementary flux vectors need not be.

To clearly highly the differences between EFMs and OFMs, we have included a toy model in the Supplementary Data. Here we provide an example of whether EFMs or OFMs are the appropriate modes for two different model setups, and detail how to calculate them in either scenario.

### 2.4 Differentiating solutions

The solution of a convex optimization problem, such as (5), with a unique optimal solution, may be implicitly differentiated (Agrawal *et al.* 2019). We previously showed that one can equate these differentials to model sensitivities (Wilken *et al.* 2022). If there are multiple optimal solutions to problem (3), a single solution must be chosen for analysis. See the Supplementary Data for a discussion on this. To ensure that differentiation is well-defined, we describe a technique for obtaining unique optimal solutions.

Definition 3 (Inactive reaction/enzyme). *In an optimal solution, an inactive reaction is one carrying zero flux; an inactive enzyme has zero concentration.*

Definition 4 (Pruned model). *A model produced by removing all reactions and enzymes of an ecGSMM that are inactive in some optimal solution of that ecGSMM is called a pruned model. Removing these reactions and enzymes is called pruning.*

Note that we also reverse any backwards reactions so that a solution to a pruned model will only have forward fluxes. In order to prove the main result of this section, that EFMs and OFMs can be implicitly differentiated, we must first discuss several necessary lemmas and show that the results for homogeneous problems (1) also apply to inhomogeneous problems (3).

Lemma 1. *The extreme rays of problem*  (5)  *with a non-zero* $\bar{v}$  *correspond to the optimal flux modes (OFMs) of*  (3).

The proof follows from the result of Gagneur and Klamt (2004) that extreme rays of a pointed polyhedral cone $\text{FC}(S)=\{x|\mathrm{Sx}=0\;,x_{i}\geq 0\}$ correspond to elementary flux modes of the homogeneous problem (1). See the Supplementary Data for details.

De Groot *et al.* (2019) previously proved that a homogeneous problem (1) with *K* enzymatic constraints will use at most *K* EFMs in an optimal solution. We extend this result to inhomogeneous problems (3) and OFMs.

Lemma 2. *An optimal solution to problem*  (3)  *with K enzymatic constraints (C4), rescaled to be in the form*
 

(7) $$C_{\Sigma }^{\left(k\right)}:=\sum_{j=1}^{r}w_{j}^{\left(k\right)}e_{j}\leq 1\;\;\text{for}\;\;k\in \{1,\ldots ,K\}$$

*will use at most K optimal flux modes.*

In short, we know from De Groot *et al.* (2019) that in the homogeneous problem (1), at most *K* EFMs will be active in an optimal solution. Combining this with Lemma 1, we prove that an optimal solution to (3) will use at most *K* OFMs in the Supplementary Data.

In practice, GSMMs always incorporate fixed fluxes, whether these are an ATP maintenance reaction or the measured exchange of a carbon source. We have proven that, equivalently to EFMs, the number of OFMs in an optimal solution is bounded by the number of active enzyme constraints. Therefore, all following results apply to EFMs and OFMs, but for brevity we use only the term OFMs unless specifying is necessary.

### 2.5 A pruned optimal solution is unique

To assert the validity of pruning models to calculate sensitivities, we state and prove conditions under which implicit differentiation is possible.

Theorem 1. *Given a model of the form*  (1)*, let us assume that all metabolic reactions have an associated enzyme, and thus an associated enzyme cost. Then, pruning an optimal solution will give a model with a unique optimal solution.*

Following the approach of De Groot *et al.* (2019), we define a cost matrix of the EFMs in a solution, and rewrite the optimization problem (1) as a maximization of EFM usage, subject to constraints relating to the cost matrix. In a genome-scale metabolic model, the enzyme turnover numbers and gene product molar masses can be assumed to have enough variation that the vectors of the cost matrix are linearly independent. This linear independence is exploited to prove that there is a unique optimal solution to a pruned model. See Supplementary Data for details.

In the setup where we have inhomogeneous constraints (3), we find again that the pruned solution is unique:

Corollary 1. *The optimal solution of a pruned ecGSMM of the form*  (3)  *is always unique.*

Proof:

We can rewrite problem (3) as problem (5), and then use the proof of Theorem 1, equating EFMs in Theorem 1 with OFMs in the current problem. ▪

Corollary 2. *Pruned models in the form of*  (1)  *or*  (3)  *can be implicitly differentiated to calculate the sensitivity of all solution variables to all model parameters.*

Proof:

The solution map of a convex optimization problem, with a unique optimal solution, may be implicitly differentiated, as proven by Agrawal *et al.* (2019). Theorem 1 and Corollary 1 ensure that pruned models do indeed have unique optimal solutions. Therefore, these solutions are also implicitly differentiable, using the method of Wilken *et al.* (2022). ▪

Corollary 2 is required to guarantee that the strategy from Wilken *et al.* (2022) is indeed generally applicable. Implicit differentiation provides a robust, efficient method for calculating solution sensitivities. Specifically, we differentiate through the Karush–Kuhn–Tucker (KKT) conditions of the optimization problem to calculate $\frac{\partial v_{i}}{\partial p_{j}}$, the sensitivity of flux through reaction *i* to parameter *j*.

Beyond the sensitivities of variables in the optimal solution, we wish to investigate the sensitivity of OFM usage to model parameters. Since OFM usage can be taken as another model variable, we may apply Corollary 2. Below, we describe the implementation of implicit differentiation for OFM sensitivities.

## 3 Materials and methods

### 3.1 Sensitivity of OFM usage to model parameters

We use implicit differentiation to robustly and efficiently calculate the sensitivities of OFM usage to model parameters. In a problem of the form (1) or (3), with *K* enzymatic constraints, we can use Lagrange multipliers to implicitly differentiate the usage of EFMs or OFMs. The full details are provided in the Supplementary Data.

### 3.2 Software implementation

To investigate OFM sensitivities, we must first calculate the OFMs in an optimal solution. To this end, we implemented the Double Description algorithm described by Terzer (2009), as well as the implicit differentiation of OFMs, in the Julia package ElementaryFluxModes.jl.

Whole solution sensitivity analysis is provided in the software package DifferentiableMetabolism.jl. This was written to be used alongside COBREXA.jl (Kratochvíl *et al.* 2025). The implementation is not limited to the ecGSMM model formulation presented in this work; it can also be applied to community models, constrained allocation flux balance analysis models (Mori *et al.* 2016), and other linear programming problems, provided a unique optimal solution can be guaranteed.

### 3.3 Models

#### 3.3.1 Enzyme-constrained fungi models

Using 342 published fungal GSMMs (Li *et al.* 2022), we made ecGSMMs to demonstrate the applicability of our software to a large number of models with no individual adjustments required. We allowed for unlimited uptake of glucose and oxygen, but constrained the total protein in the model to 35% of the cell dry weight, so that the models were each subject to a single enzyme constraint. We used the $k_{\text{cat}}$ prediction results provided by the same study as the models, and enzyme complexes were assumed to contain a 1:1 ratio of subunits. All metabolic reactions without a gene-reaction-rule were given a proxy enzyme with the average $k_{\text{cat}}$ and gene product molar mass of the model.

#### 3.3.2 *E. coli* enzyme-constrained metabolic model

The iML1515 model of *E. coli* MG1655 (Monk *et al.* 2017) was used as the base GSMM. Enzyme turnover numbers were taken from Heckmann *et al.* (2020). If available, enzyme subunit stoichiometries were taken from the complex portal (Meldal et al. 2015), otherwise Swiss-Prot entries (The UniProt Consortium 2025) were used. If no information was available, enzyme complexes were assumed to contain a 1:1 ratio of subunits. The model was given the enzyme capacity constraints of $160\frac{\text{mg}}{g_{\text{DCW}}}$ membrane protein, and $340\frac{\text{mg}}{g_{\text{DCW}}}$ in the rest of the cell. The numbers used for these bounds were taken from absolute quantitative proteomics measurements with glucose as the sole carbon source (Schmidt *et al.* 2016).

Acetate production was investigated by using the sensitivities of two OFMs in the optimal solution of the ecGSMM of iML1515. Experimental effects of gene knockouts on acetate production were taken from the measurements in Contiero *et al.* (2000), Zhao *et al.* (2004), De Maeseneire *et al.* (2006), Fong *et al.* (2006), Long *et al.* (2016), Lozano Terol *et al.* (2019).

## 4 Results

### 4.1 Software performance

The software presented in this paper allows for fast differentiation of model solutions to model parameters. We have evaluated the computational resources required for DifferentiableMetabolism.jl to calculate the sensitivity of all model variables with respect to all model parameters, and compared this to the time taken by a trivial implementation of central finite differencing. Our implementation of central finite differencing involved perturbing a parameter 0.1% above and 0.1% below the baseline value, and calculating the slope of the change in model variables as an approximation for the sensitivity of variables to parameters. Conversely, implicit differentiation is mathematically exact, and accuracy only relies only on the robustness of numerically solving a system of linear equations.

In the benchmark settings, our software was around 4× more efficient than finite differencing (Table 1) on full models, and more than 25× more efficient with OFM-based sensitivity calculations. Notably, with finite differencing the OFMs of the pruned models need to be recalculated for each parameter perturbation. This is required to compensate for changes in the optimal objective caused by the perturbations, which invalidate the calculated OFM. DifferentiableMetabolism.jl only requires that the OFMs are calculated once; which is followed by solving a single system of linear equations that yields all sensitivities. The practically applicable efficiency improvement, comparing the better settings for each method, was around 7× in the benchmark.

**Table 1.** Calculation of sensitivities more efficient using DifferentiableMetabolism.jl (labelled DiffMet in the table) than using central finite differencing (CDiff)^a^.

			CPU time consumed (s)	
Repr.	Model	#vars	DiffMet	CDiff
Full	yeastGEM	436	7.48 ± 0.11	32.45 ± 0.32
	iML1515	394	6.24 ± 0.08	27.06 ± 0.31
OFMs	yeastGEM	2	3.81 ± 0.15	144.90 ± 0.99
	iML1515	2	3.87 ± 0.19	99.58 ± 0.77

a We report the single-CPU-core time as the geometric mean ± standard deviation of 10 replicate simulations, for the sensitivity of all variables in the optimal model solutions, as well as of the OFM usage in the optimal solutions, to all parameters. All simulations in this paper were run on an AMD Ryzen 9 5950X with 32 GB main memory.

### 4.2 Sensitivity analysis of 342 fungal models

In order to demonstrate the robustness and scalability of DifferentiableMetabolism.jl, we simulated growth of 342 fungal models and calculated the sensitivity of their growth rates to the model parameters. Using traditional finite difference techniques would require solving at least 342×n linear programs, where *n* denotes the number of parameters in each model. Using our software, we only needed to solve one system of linear equations per model (after the initial LP was solved). In turn, our routine was more efficient than using finite differencing.

**Figure 1.**
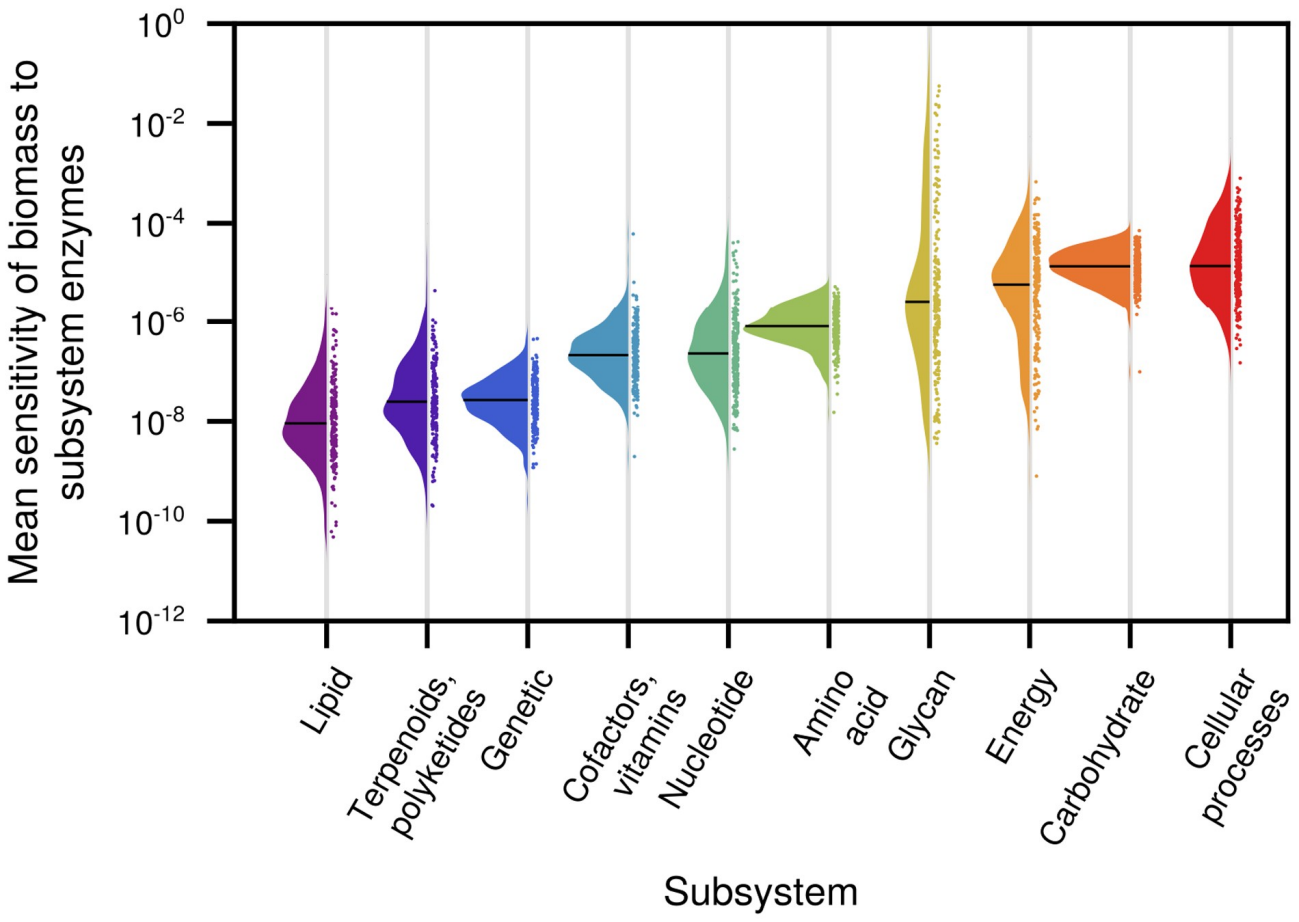
The mean sensitivity of biomass reactions across 342 fungal species is consistent across all subsystems except glycan. Each point on the plot is the mean sensitivity of the growth of a model to its enzyme turnover numbers in a subsystem. Rain clouds show the spread across sensitivity of model growth rates to kinetic parameters in one subsystem.

Figure 1 summarizes the variation of growth sensitivity to model reaction subsystems. Each point gives the mean sensitivity of the growth of a model to the $k_{\text{cats}}$-values within a subsystem. A possible application of our framework is in metabolic engineering: interestingly, we observed a large spread in the sensitivity of growth to glycan metabolism across the models. Glycan engineering in yeast species focuses on the production of proteins carrying human-compatible glycosylation (Piirainen *et al.* 2022). Deciding on which organisms to focus engineering efforts requires, amongst other factors, knowledge of the impact of metabolic alterations on the growth.

Our results indicate that the response of yeast species to changes in $k_{\text{cat}}$-values in glycan metabolism varies widely. The models with growth predicted to be least sensitive to glycan metabolism include *Saccharomyces cerevisiae, Lachancea fermentati, Hanseniaspora valbyensis*, and *Kazachstania naganishii*, which are all organisms that have been successfully used in glycosylation production (Chiba and Akeboshi 2009), or proposed or used for other bio-processes (Bellut *et al.* 2020, Liu *et al.* 2021, Abd-El-Haleem *et al.* 2024).

### 4.3 OFM sensitivity predictions of acetate production in *E. coli*

We observed that sensitivities of OFMs captured gene knockout effects better than performing standard *in silico* gene knockouts. Since *E. coli* is one of the best characterized and most successfully utilized organisms in biotechnology, we attempted to validate the OFM sensitivities with 23 known *E. coli* knockouts of 16 different genes, where some genes have been knocked out in more than one study. It is a recurring theme in *E. coli* engineering to reduce the formation of acetate (De Mey *et al.* 2007), since its presence can slow down growth (Nakano *et al.* 1997) and decrease product yield (Pan *et al.* 1987). We compared the phenotype in 23 *E. coli* knockout mutants to the sensitivity of an acetate producing OFM to the associated parameters.

As expected from Theorem 2, we obtained a superposition of two OFMs; an OFM composed of respiratory pathways that produces 0.05 mol of acetate per mol of glucose, and an aero-fermentative OFM producing 0.36 mol acetate per mol glucose.

**Figure 2.**
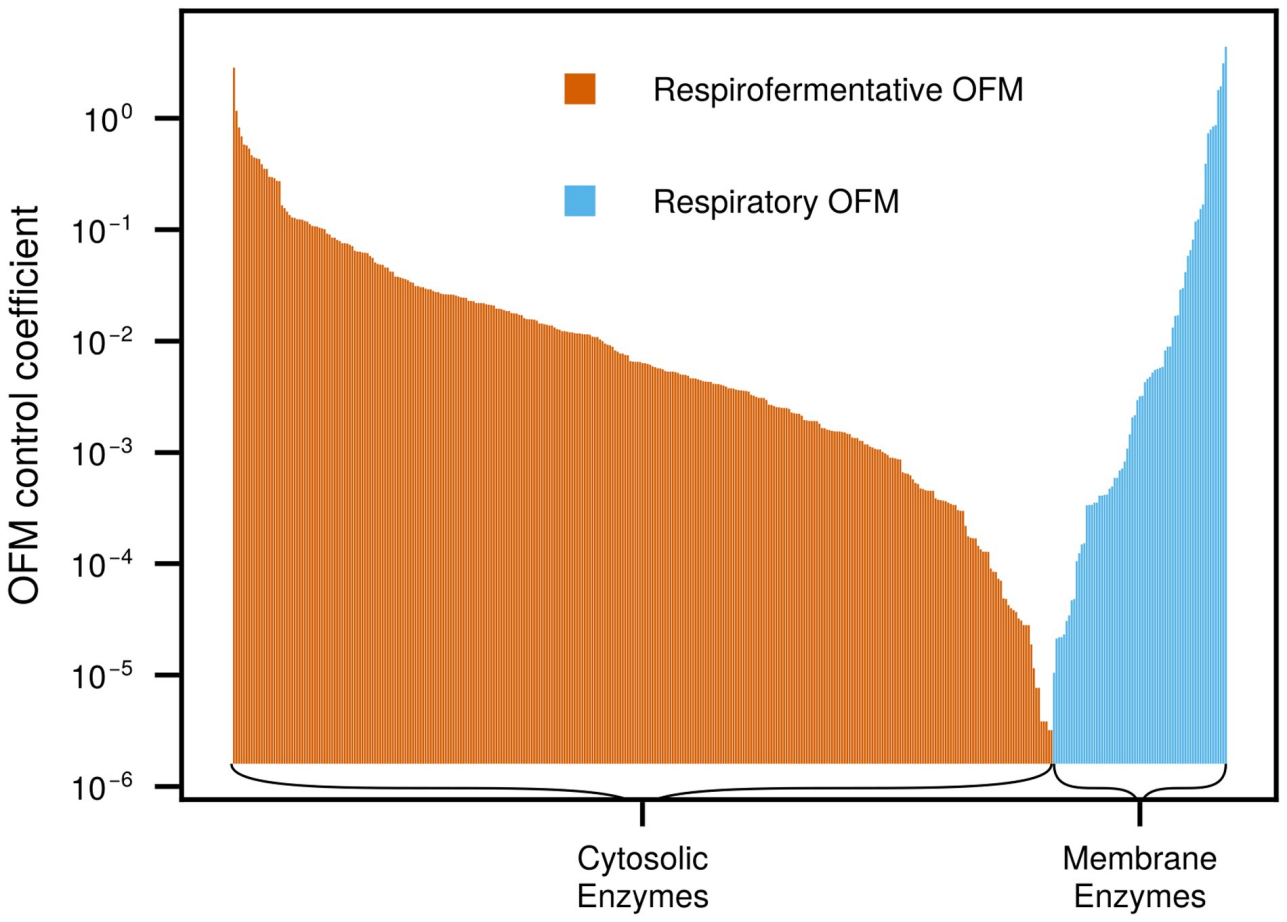
Control coefficients of OFM usage in *E. coli*, $\frac{p}{\lambda }\frac{\partial \lambda }{\partial p}$, support the assumption that space in the membrane controls respiration. Along the x-axis we have every enzyme parameter, *p*, in the active solution, and we plot on the y-axis whichever of $\frac{p}{\lambda_{1}}\frac{\partial \lambda_{1}}{\partial p}$ and $\frac{p}{\lambda_{2}}\frac{\partial \lambda_{2}}{\partial p}$ is greater than zero. Increasing any kinetic parameter in the cytosol will increase the use of the respirofermentative OFM (orange), and decrease the use of the respirative OFM (blue), in the optimal solution. The opposite is true for any membrane bound kinetic parameter.

Figure 2 shows that an increase in the turnover number of any membrane enzyme also increases the usage of the respiratory OFM, which in turn decreases the usage of the aero-fermentative OFM. The opposite effect is observed with cytosolic enzymes: major components of the respiratory pathway, including oxidative phosphorylation, are located in the cytoplasmic membrane. Therefore, if any enzyme in the membrane, including non-respiratory enzymes, has its turnover number increased, a smaller concentration of this enzyme will be required to catalyse the same flux (recall that $v=k_{\text{cat}}\cdot e$) and there will hence be more available space in the membrane for every other membrane bound enzyme.

**Figure 3.**
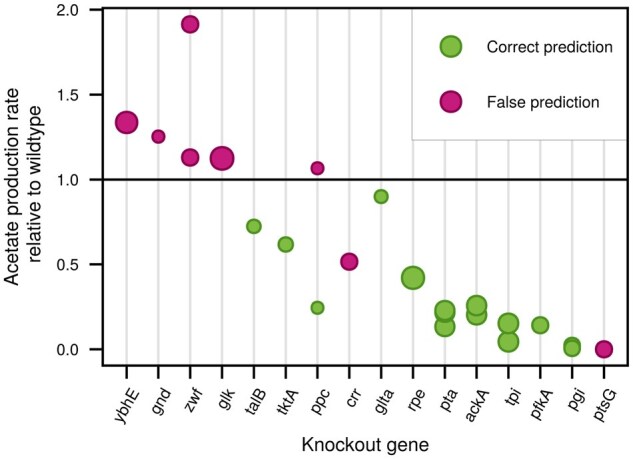
Sensitivity of the respirofermentative OFM in *E. coli* provides qualitatively accurate predictions of those gene knockouts that decrease acetate production. We plot the acetate production rate in 23 knockout mutants of 16 different genes relative to the wildtype, taken from experimental data (see Section 3). The size of the marker corresponds to the size of the control coefficient $\frac{p_{ko}}{\lambda_{ac}}\frac{\partial \lambda_{ac}}{\partial p_{ko}}$ of the acetate producing OFM, $\lambda_{ac}$, to the turnover number, $p_{ko}$, of the enzyme of the knockout gene. The colour indicates whether this prediction matches the experimentally observed change in acetate in a knockout mutant.

In Fig. 3, we see that for 15 of a total 23 gene knockouts, experimental increases in acetate production were correctly predicted by the sensitivity of the acetate producing OFM (shown in green). We considered a prediction correct either if, upon knocking out the gene, acetate production decreases and the sensitivity of the OFM to the $k_{\text{cat}}$ is positive, or if acetate production increases and the sensitivity is negative. A positive sensitivity implies that OFM use increases or decreases together with the parameter change, negative sensitivity implies opposite direction of changes.

We further analysed the reasons for prediction failures in the eight knockouts. The majority (six out of eight) of the incorrect predictions were from knockout mutants where the acetate production increased compared to the wildtype. A possible additional discrepancy in two experiments has caused a prediction failure of sensitivity for phosphoenolpyruvate carboxylase (ppc) as seen in Fig. 3, because the experiments measured opposite effects. Clarification of this problem might require additional reproduction of the experiments with ppc.

We hypothesize that pruning of the unused pathways before calculating the sensitivities might have caused some of the incorrect sensitivities. Pruned models capture local (infinitesimal) changes in turnover numbers, but cannot capture larger metabolic switches that might occur in the greater neighbourhood of the given flux mode. It is noteworthy that despite this drawback, only one-third of the knockouts actually had an effect different to the sensitivity of the OFM.

A further potential reason for OFM sensitivity failing to predict knockout effects is that the iML1515 model could simply be missing (or erroneously including) some reactions. To test this hypothesis, we performed *in silico* gene knockouts with the standard GSM iML1515 without any enzyme constraints. We found that for 6 of the total 16 genes, the model either predicted no growth or an incorrect change in acetate production, thus performing worse than our OFM analysis.

## 5 Discussion

In this study, we have proven that pruning models leaves a unique optimal solution, which enables implicit differentiation to calculate sensitivities of the typical variables (flux rates, enzyme concentrations) and the usage of the OFMs. Many methods for the sensitivity analysis of constraint-based models analyse only the sensitivity of growth to parameters. This is of course an important factor, but neglects a fine-grained view that is prospectively useful, such as by-product sensitivities in metabolic engineering. The method introduced by Wilken *et al.* (2022), and implemented here with DifferentiableMetabolism.jl, robustly and efficiently calculates the sensitivity of all model variables (fluxes and enzyme concentrations) to all model parameters (kinetic constants, upper and lower bounds on fluxes, and capacity constraints). This allows for a much wider analysis of the metabolism of an organism, as demonstrated by our analysis of acetate production in *E. coli* knockout mutants. Moreover, control of model parameters can be holistically summarized by changes in OFMs, which provide a more interpretable overview of the active metabolic routes than a full-scale sensitivity analysis of every model variable.

The sensitivity of growth to model parameters is used to highlight the subsystems whose reactions have a large effect on growth in published fungal models. We found that the models whose growth was least sensitive to parameters of glycan metabolism have indeed been used either in glycosylation processes, or for other general metabolic engineering purposes. This validates our method as a useful tool in screening for potential microbial cell factories, and showcases DifferentiableMetabolism.jl as a scalable and robust tool to be used in future for general-purpose sensitivity analysis.

Our use of the OFM sensitivity to predict gene knockout effects in *E. coli* gave qualitatively more accurate predictions than *in silico* gene knockout experiments of the iML1515 GSMM. However, the predictions did not fully match the experimental data. We assume that such analysis would be better suited to simulation of knockdowns or over-expression, but such validation is challenging due to a current lack of experimental data, and the high cost of these measurements.

Our work highlights that novel analysis techniques, such as OFM sensitivities, are of benefit when investigating model behaviour. We provide a robust, fast, and generally applicable scheme through which to analyse the control of model parameters on the whole solution, in contrast to previous efforts that only considered a simplified view of growth sensitivity. The analysis of OFMs in an optimal solution helps to provide a holistic understanding of the effects of local changes on global phenotypes. The pruning method assumes that infinitesimal parameter changes do not cause switching between optimal solutions. An analysis into the switching points of metabolism would be invaluable in future work.

Our performance measurements have provided a strong indication that the method is sufficiently efficient to scale to larger or community-wide models. Since both DifferentiableMetabolism.jl and the finite differencing algorithm offer possibilities for acceleration via parallel hardware, we aim to evaluate and compare their optimized parallel implementations in the future, giving a suitable guideline for high-performance analysis of the model sensitivities.

Supported by the new software packages presented in this paper, we expect the differentiation of model solutions to be increasingly used to investigate microbial metabolism.

## Supplementary Material

btaf287_Supplementary_Data

## Data Availability

Software introduced here is available as open-source Julia packages DifferentiableMetabolism.jl (https://github.com/stelmo/DifferentiableMetabolism.jl) and ElementaryFluxModes.jl (https://github.com/HettieC/ElementaryFluxModes.jl), which both work on all major operating systems and computer architectures. Code to reproduce all results is available from https://github.com/HettieC/DifferentiableOFMPaper, and as an archive from https://doi.org/10.5281/zenodo.15183208.
