# Supplementary material for: Algebraic differentiation for fast sensitivity analysis of optimal flux modes in metabolic models
Source: Bioinformatics. 2025 May 6;41(6):btaf287. doi: 10.1093/bioinformatics/btaf287 (PMC12133274; doi:10.1093/bioinformatics/btaf287)
Supplement: btaf287_Supplementary_Data [file btaf287_supplementary_data.pdf]

Supplementary Material

S1 Toy model

In this section, we provide a simplified enzyme-constrained metabolic model (Figure S1), to demonstrate pruning optimal solutions, calculating OFMs, and to highlight the difference between EFMs and OFMs. We constrain the model with two distinct enzyme pools. We give reactions R_3 to R_5 appropriate k_{cat} values and gene product molar masses to ensure that both R_5 and reactions R_3 and R_4 are used in the optimal solution, but R_8 is not.

To demonstrate calculation of EFMs in an optimal solution, the LP is set up as follows:

$$\begin{aligned} \text{maximise} \quad & v_6 \\ \text{subject to} \quad & \text{(C1)}\ \ \boldsymbol{Sv} = \boldsymbol{0} \\ & \text{(C2)}\ \ v_i \geq 0\ \ \forall i \\ & \text{(C3)}\ \ v_i = k_{\text{cat},i} \cdot e_i\ \ \forall i \in [3,4,5] \\ & \text{(C4)}\ \ e_1 + e_2 \leq E_1 \\ & \text{(C5)}\ \ e_3 + e_4 + e_5 \leq E_2 \end{aligned} \tag{S1}$$

When we optimise, we get the solution in Figure S1e, and can see that reaction R_7 and R_8 have zero flux. Therefore, these two reactions are pruned from the model, and we are left with the network in Figure S1b and the stoichiometric matrix in S1d. When we then optimise the LP in (S1), using this pruned stoichiometric matrix $\boldsymbol{S}$, we find the optimal solution in S1f. By theorem 1, we know that this is a unique optimal solution, and thus calculate the EFMs given in S1g. In S1h, we give the usage of the two EFMs in the optimal solution.

Adding the condition (C6), $v_7 = 15$, to the LP in (S1), we ensure that reaction R_7 carries flux. This is included to demonstrate how we would treat a reaction in a genome-scale model with an active inhomogeneous flux constraint or a known flux, for example a fixed glucose exchange or an ATP maintenance reaction. We now no longer have a flux cone but rather a flux polyhedron. We therefore need to calculate OFMs, rather than EFMs. First, we solve the LP, and see in Figure S2e that reaction R_8 is now the only reaction carrying zero flux. Pruning the model removes R_8, and when we resolve the LP with the pruned stoichiometric matrix from S2d, we once again find a unique optimal solution.

The difference between how to calculate OFMs and EFMs now becomes apparent. After pruning, the first step is to separate the pruned stoichiometric matrix into $\boldsymbol{S}^{(1)}$ and $\boldsymbol{S}^{(2)}$, the columns corresponding to unfixed and fixed reactions, respectively. In this case, we know that $v_7 = 15$ and the objective $v_6 = 60$ in the optimal solution. Therefore, reactions R_6 and R_7 are taken into $\boldsymbol{S}^{(2)}$. All other fluxes are variables, and will be taken into $\boldsymbol{S}^{(1)}$, as seen in Figure S3b and S3a. Next, we need to multiply $\boldsymbol{S}^{(2)}$ by $\boldsymbol{v}^{(2)} = \begin{bmatrix} v_6 & v_7 \end{bmatrix}'$ to use this in the matrix $\boldsymbol{A}$ (S3d), creating a slack variable. Running the double description algorithm, we calculate the extreme rays of the optimal flux cone $\mathbf{OFC}(\boldsymbol{A}) = \{\boldsymbol{x} \in \mathbb{R}^n | \boldsymbol{Ax} = \boldsymbol{0}, v_i \geq 0\}$ as in Figure S3e. The last row corresponds to the slack variable introduced into the last column of $\boldsymbol{A}$. If required, we rescale the extreme rays so

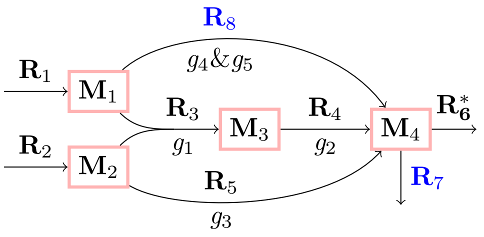

(a) Model network

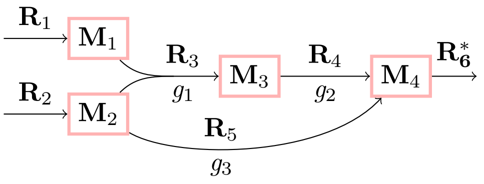

(b) Pruned network

$$\boldsymbol{S} = \begin{bmatrix} 1 & 0 & -1 & 0 & 0 & 0 & 0 & -1 \\ 0 & 1 & -1 & 0 & -1 & 0 & 0 & 0 \\ 0 & 0 & 1 & -1 & 0 & 0 & 0 & 0 \\ 0 & 0 & 0 & 1 & 1 & -1 & -1 & 1 \end{bmatrix}$$

(c) Full stoichiometric matrix

$$\boldsymbol{S} = \begin{bmatrix} 1 & 0 & -1 & 0 & 0 & 0 \\ 0 & 1 & -1 & 0 & -1 & 0 \\ 0 & 0 & 1 & -1 & 0 & 0 \\ 0 & 0 & 0 & 1 & 1 & -1 \end{bmatrix}$$

(d) Pruned stoichiometric matrix

$$\boldsymbol{v}^* = \begin{bmatrix} v_1 \\ v_2 \\ v_3 \\ v_4 \\ v_5 \\ v_6 \\ v_7 \\ v_8 \end{bmatrix} = \begin{bmatrix} 50 \\ 75 \\ 50 \\ 50 \\ 25 \\ 75 \\ 0 \\ 0 \end{bmatrix}$$

(e) Optimal solution (full model)

$$\boldsymbol{v}^* = \begin{bmatrix} v_1 \\ v_2 \\ v_3 \\ v_4 \\ v_5 \\ v_6 \end{bmatrix} = \begin{bmatrix} 50 \\ 75 \\ 50 \\ 50 \\ 25 \\ 75 \end{bmatrix}$$

(f) Optimal solution (pruned model)

$$\textbf{EFM} = \begin{bmatrix} 1 & 0 \\ 1 & 1 \\ 1 & 0 \\ 1 & 0 \\ 0 & 1 \\ 1 & 1 \end{bmatrix}$$

(g) EFMs

$$\boldsymbol{v}^* = 50 \cdot \mathrm{EFM}_1 + 25 \cdot \mathrm{EFM}_2$$

(h) Proportions of EFMs in optimal solution

Figure S1: Procedure to calculate the EFMs in the network given in (a). Reactions shown in blue have zero flux in the optimal solution and are pruned before calculating EFMs.

that this last row is equal to $\begin{bmatrix} 1 & 1 \end{bmatrix}$, and reintroduce R_6 and R_7 into their original positions and with their original fixed values. The result of this is the final OFMs, given by the two columns in Figure S3f. We see that both OFMs produce the optimal objective flux, $v_6 = 60$, and use the required $v_7 = 15$. As with EFMs, linearly scaled OFMs are equivalent, so we scale to have flux through the objective R_6 equal to 1 in both OFMs. The optimal solution can be decomposed into $\boldsymbol{v}^* = 40 \cdot \mathrm{OFM}_1 + 20 \cdot \mathrm{OFM}_2$.

S2 Theory

Here, we provide the mathematical proofs of all lemmas and theorems lacking a formal proof in the main text.

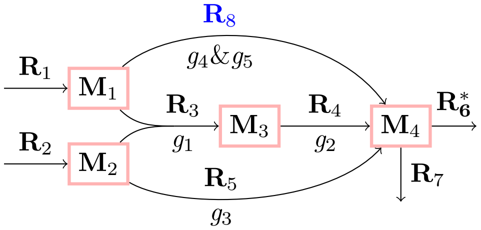

(a) Model network

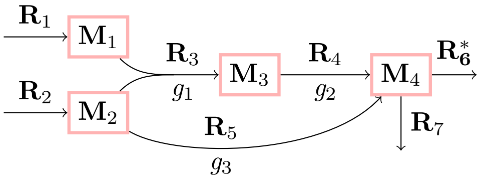

(b) Pruned network

$$\boldsymbol{S} = \begin{bmatrix} 1 & 0 & -1 & 0 & 0 & 0 & 0 & -1 \\ 0 & 1 & -1 & 0 & -1 & 0 & 0 & 0 \\ 0 & 0 & 1 & -1 & 0 & 0 & 0 & 0 \\ 0 & 0 & 0 & 1 & 1 & -1 & -1 & 1 \end{bmatrix}$$

(c) Full stoichiometric matrix

$$\boldsymbol{S} = \begin{bmatrix} 1 & 0 & -1 & 0 & 0 & 0 & 0 \\ 0 & 1 & -1 & 0 & -1 & 0 & 0 \\ 0 & 0 & 1 & -1 & 0 & 0 & 0 \\ 0 & 0 & 0 & 1 & 1 & -1 & -1 \end{bmatrix}$$

(d) Pruned stoichiometric matrix

$$\boldsymbol{v}^* = \begin{bmatrix} v_1 \\ v_2 \\ v_3 \\ v_4 \\ v_5 \\ v_6 \\ v_7 \\ v_8 \end{bmatrix} = \begin{bmatrix} 50 \\ 75 \\ 50 \\ 50 \\ 25 \\ 60 \\ 15 \\ 0 \end{bmatrix}$$

(e) Optimal solution (full model)

$$\boldsymbol{v}^* = \begin{bmatrix} v_1 \\ v_2 \\ v_3 \\ v_4 \\ v_5 \\ v_6 \\ v_7 \end{bmatrix} = \begin{bmatrix} 50 \\ 75 \\ 50 \\ 50 \\ 25 \\ 60 \\ 15 \end{bmatrix}$$

(f) Optimal solution (pruned model)

Figure S2: Pruning a model with inhomogeneous flux constraints

$$\boldsymbol{S}^{(1)} = \begin{bmatrix} 1 & 0 & -1 & 0 & 0 \\ 0 & 1 & -1 & 0 & -1 \\ 0 & 0 & 1 & -1 & 0 \\ 0 & 0 & 0 & 1 & 1 \end{bmatrix}$$

(a) Unknown flux stoichiometries

$$\boldsymbol{S}^{(2)} = \begin{bmatrix} 0 & 0 \\ 0 & 0 \\ 0 & 0 \\ -1 & -1 \end{bmatrix}$$

(b) Fixed flux stoichiometries

$$\boldsymbol{w} = \begin{bmatrix} 0 \\ 0 \\ 0 \\ -75 \end{bmatrix}$$

(c) $\boldsymbol{S}^{(2)}\boldsymbol{v}^{(2)}$

$$\boldsymbol{A} = \begin{bmatrix} 1 & 0 & -1 & 0 & 0 & 0 \\ 0 & 1 & -1 & 0 & -1 & 0 \\ 0 & 0 & 1 & -1 & 0 & 0 \\ 0 & 0 & 0 & 1 & 1 & -75 \end{bmatrix}$$

(d) Augmented matrix, $\boldsymbol{A} = \begin{bmatrix}\boldsymbol{S}^{(1)} & \boldsymbol{w}\end{bmatrix}$

$$\text{extreme rays} = \begin{bmatrix} 75 & 0 \\ 75 & 75 \\ 75 & 0 \\ 75 & 0 \\ 0 & 75 \\ 1 & 1 \end{bmatrix}$$

(e) Extreme rays of OFC($\boldsymbol{A}$)

$$\mathbf{OFM} = \begin{bmatrix} 75 & 0 \\ 75 & 75 \\ 75 & 0 \\ 75 & 0 \\ 0 & 75 \\ 60 & 60 \\ 15 & 15 \end{bmatrix} = \begin{bmatrix} 1.25 & 0 \\ 1.25 & 1.25 \\ 1.25 & 0 \\ 1.25 & 0 \\ 0 & 1.25 \\ 1 & 1 \\ 0.25 & 0.25 \end{bmatrix}$$

(f) OFMs of P($\boldsymbol{S}$)

$$\boldsymbol{v}^* = \lambda_1 \cdot \text{OFM}_1 + \lambda_2 \cdot \text{OFM}_2 = 40 \cdot \text{OFM}_1 + 20 \cdot \text{OFM}_2 \tag{S2}$$

Figure S3: Implementation in ElementaryFluxModes.jl of calculating OFMs of a pruned optimal solution.

S2.1 Optimal flux modes

Lemma 1. *The extreme rays of problem (5) with a non-zero* $\overline{v}$ *correspond to the optimal flux modes (OFMs) of (3).*

Proof. : Previously, Gagneur Gagneur and Klamt (2004) proved that extreme rays of a pointed polyhedral cone
FC($\boldsymbol{S}$) $= \{\boldsymbol{x}|\boldsymbol{S}\boldsymbol{x} = \boldsymbol{0}\ , x_i \geq \boldsymbol{0}\}$ correspond to the elementary flux modes of the problem (1).

We define the pointed polyhedral cone FC($\boldsymbol{A}$) $= \{\boldsymbol{x}|\boldsymbol{A}\boldsymbol{x} = \boldsymbol{0},\ \boldsymbol{x} \geq \boldsymbol{0}\}$, where $\boldsymbol{A} := \begin{bmatrix}\boldsymbol{S^{(1)}} & \boldsymbol{w}\end{bmatrix}$ as in problem (5). The cone FC($\boldsymbol{A}$) is in the same form as FC($\boldsymbol{S}$), where $\boldsymbol{S}$ is the stoichiometric matrix of (1). Thus, the extreme rays of FC($\boldsymbol{A}$) correspond to the elementary flux modes of $\boldsymbol{A}$ Gagneur and Klamt (2004).

Elementary flux modes, and optimal flux modes, are the elementary modes of the associated polyhedral cone. An EFM or OFM producing one unit of objective flux need not adhere to enzyme constraints, since the optimal solution will use a scaled sum of the EFMs or OFMs. Thus, we can always scale an extreme ray $\boldsymbol{x} = \begin{bmatrix}\boldsymbol{v^{(1)}} & \overline{v}\end{bmatrix}$ of FC($\boldsymbol{A}$) so that $\overline{v} = 1$. Therefore, all vectors $\boldsymbol{x}$ admissible in FC($\boldsymbol{A}$), with a non-zero $\overline{v}$, must carry flux in the optimal ratio of the objective to the fixed fluxes.

To illustrate this optimal ratio, let $\boldsymbol{x}$ be the vector of fluxes $\boldsymbol{x} = [v_1, \ldots, v_r, ;\overline{v}]$, where $v_1 = c_1$ is a fixed known flux and v_r is the objective flux, with optimal value c_r. All admissible vectors in FC($\boldsymbol{A}$) must obey $v_1/v_r = c_1/c_r$. Therefore, as extreme rays of FC($\boldsymbol{A}$) give the elementary flux modes of A, these extreme rays equivalently correspond to the optimal flux modes of (3).

□

Lemma 2. *An optimal solution to problem (3) with K enzymatic constraints (C4), rescaled to be in the form*

$$C_{\Sigma}^{(k)} := \sum_{j=1}^{r} w_j^{(k)} e_j \leq 1 \;\; \textit{for} \;\; k \in \{1, ..., K\} \tag{S3}$$

will use at most K optimal flux modes.

Proof. :

Firstly, we know that extreme rays of the homogeneous polyhedral cone $\mathrm{FC}(\boldsymbol{S}) = \{\boldsymbol{v} | \boldsymbol{S}\boldsymbol{v} = \boldsymbol{0},\ \boldsymbol{v} \geq \boldsymbol{0}\}$ for a stoichiometric matrix $\boldsymbol{S}$ from problem (1) correspond to the elementary flux modes Gagneur and Klamt (2004). We also know that an optimal solution to (1) with K enzymatic constraints in the form (S3) will use at most K EFMs De Groot et al. (2019). Therefore, since extreme rays of $\mathrm{FC}(\boldsymbol{S})$ correspond to EFMs of (1), there will be at most K extreme rays used in an optimal solution to $\mathrm{FC}(\boldsymbol{S})$ with K linear enzymatic constraints.

Now we consider problem (3). From Lemma 1, we know that extreme rays with non-zero $\overline{v}$ of the polyhedral cone $\mathrm{FC}(\boldsymbol{A})$ where $\boldsymbol{A} = \begin{bmatrix} \boldsymbol{S^{(1)}} & \boldsymbol{w} \end{bmatrix}$ correspond to optimal flux modes of the problem (3).

We can transform the inhomogeneous problem (3) with K enzymatic constraints into the form (5), and we obtain a polyhedral cone $\mathrm{FC}(\boldsymbol{A}) = \{\boldsymbol{v} | \boldsymbol{A}\boldsymbol{v} = \boldsymbol{0},\ \boldsymbol{v} \geq \boldsymbol{0}\}$ where $\boldsymbol{A} = \begin{bmatrix} \boldsymbol{S^{(1)}} & \boldsymbol{w} \end{bmatrix}$. We may now regard $\boldsymbol{A}$ in the same way as the above $\boldsymbol{S}$, and see that an optimal solution to $\mathrm{FC}(\boldsymbol{A})$ with K enzymatic constraints uses at most K extreme rays. The extreme rays of $\mathrm{FC}(\boldsymbol{A})$ with non-zero $\overline{v}$ correspond to the optimal flux modes of (3), and thus an optimal solution will use at most K OFMs.

□

S2.2 A pruned optimal solution is unique

To assert the validity of pruning models to calculate sensitivities, we prove conditions under which implicit differentiation is possible.

Theorem 1. *Given a model of the form (1), let us assume that all metabolic reactions have an associated enzyme, and thus an associated enzyme cost. Then, pruning an optimal solution will give a model with a unique optimal solution.*

Proof. : Let the objective of the LP (1) be to maximise the flux through reaction r. We first prove the simplest case where there is only one capacity constraint.

Assume that the model has one capacity constraint in the form $C_{\Sigma} = \sum_{j=1}^{r} w_j e_j \leq 1$, where w_j is the fraction of the enzyme pool that one mole of the j-th enzyme uses up. In this case, if the problem is solvable then any solution will be composed of one EFM De Groot et al. (2019). Pruning the model will thus leave only the reactions that are in this EFM. Maximising the objective will leave no flux variability, since knowing the flux through one reaction in an EFM determines the flux through every other reaction. There will also be no variation in the enzyme concentrations if the turnover numbers and masses are sufficiently different, which we can assume is the case in a full-scale ecGSMM. As required, we thus have a unique optimal solution in a pruned model with one capacity constraint.

It now remains to prove that a pruned model with $K > 1$ capacity constraints has a unique optimal solution. Following the approach of de Groot et al. De Groot et al. (2019), if we have K active enzyme constraints, then we have $C_{\Sigma}^{(k)} = \sum_{j=1}^{r} w_j^k e_j^k = 1$ for every constraint k, where e_j^k gives the concentration of enzyme j in EFM k. We have at most K EFMs in our optimal solution. The flux through reaction i in EFM k is denoted V_i^k. Each EFM is then rescaled such that the flux

through the objective reaction, r, through each EFM k, denoted V_r^k, is equal to 1: $\mathbf{EFM}^k = (V_1^k, \ldots, V_{r-1}^k, 1)$. The flux solution can now be written as a linear combination of these EFMs:

$$\boldsymbol{v} = \lambda_1 \mathbf{EFM}^1 + \ldots + \lambda_K \mathbf{EFM}^K, \text{ where } \lambda_k \geq 0 \quad \forall\, 1 \leq k \leq K \tag{S4}$$

The quantity of objective flux in the optimal solution contributed by the k-th EFM is denoted by λ_k.

The fluxes V_j^k through each EFM are fixed, since knowing one flux in an EFM fully determines every other reaction flux. It now remains to show that the λ_i are also fixed.

Since $v_i = k_{cat,i} \cdot e_i$, the enzyme constraints can be written as

$$\begin{aligned} 1 = C_\Sigma^{(k)} &= \sum_{j=1}^{r} w_j^k e_j^k \\ &= \sum_{j=1}^{r} w_j^k \sum_{i=1}^{K} \lambda_i \frac{V_j^i}{k_{cat,j}} \\ &= \sum_{i=1}^{K} \lambda_i \sum_{j=1}^{r} w_j^k \frac{V_j^i}{k_{cat,j}} \\ &= \sum_{i=1}^{K} \lambda_i \sum_{j=1}^{r} d_k^i \end{aligned} \tag{S5}$$

where $d_k^i := \sum_{j=1}^{r} w_j^k \frac{V_j^i}{k_{\mathrm{cat},j}}$ denotes the total protein cost to constraint pool k of one unit of objective flux through $\mathbf{EFM}^i$.

From equation (S4), the objective flux v_r can be written as

$$v_r = \lambda_1 + \ldots + \lambda_K \tag{S6}$$

meaning we can rewrite the optimisation as a maximisation of the sum of the λ_i, that must adhere to the constraint given by (S5):

$$\max_{\boldsymbol{\lambda}} \{ \sum_{i}^{K} \lambda_i : \quad \lambda_i \geq 0, \quad \boldsymbol{D\lambda} \leq 1 \} \tag{S7}$$

where $\boldsymbol{D} = [\boldsymbol{d^1} \ldots \boldsymbol{d^L}]$.

We now build upon the work of de Groot et al. De Groot et al. (2019) to prove that pruning the model does indeed leave a unique optimal solution.

Due to the setup of the problem, an optimal solution with $K > 1$ enzyme constraints will use $L \leq K$ EFMs, and these EFMs will always satisfy $\lambda_1 + \cdots + \lambda_L = v_r$. Therefore, we can always write the linear system of equations

$$\begin{bmatrix} \mathbf{1} \\ \boldsymbol{D} \end{bmatrix} \begin{bmatrix} \lambda_1 \\ \vdots \\ \lambda_L \end{bmatrix} = \begin{bmatrix} v_r \\ \mathbf{1} \end{bmatrix} \tag{S8}$$

where $\begin{bmatrix} \mathbf{1} & \boldsymbol{D} \end{bmatrix}^T$ is a $(K+1) \times L$ matrix. The system (S8) has a unique solution when $\begin{bmatrix} \mathbf{1} & \boldsymbol{D} \end{bmatrix}^T$ has rank L, which is the case if the L cost vectors of the L EFMs are linearly independent. Where there is difference in enzyme costs, the cost vectors will be linearly independent, which implies that

the solution to (S7) will be unique. Thus, the optimal solution of a pruned model in the form (1) is unique.

□

In the setup where we have inhomogeneous constraints (3), we find again that the pruned solution is unique:

Corollary 1. *The optimal solution of a pruned ecGSMM (3) is always unique.*

Proof. : We can rewrite problem (3) as problem (5), and then use the proof of Theorem 1, equating EFMs in Theorem 1 with OFMs in the current problem.

□

Corollary 2. *Pruned models in the form of (1) or (3) can be implicitly differentiated to calculate the sensitivity of all solution variables to all model parameters.*

Proof. : The solution map of a convex optimisation problem, with a unique optimal solution, may be implicitly differentiated, as proven by Agrawal et al. Agrawal et al. (2019). Theorem 1 and Corollary 1 ensure that pruned models do indeed have unique optimal solutions. Therefore, these solutions are also implicitly differentiable, using the method of Wilken et al. Wilken et al. (2022).

□

S2.3 Differentiating OFM usage

In order to differentiate OFM usage, we reformulate problem (5) of maximising the objective flux subject to enzyme constraints, as a problem of maximising the sum of the OFM usage subject to equivalent constraints:

Let $\boldsymbol{p}$ be the vector of model parameters, and $\boldsymbol{\lambda}(\boldsymbol{p}) = [\lambda_1(\boldsymbol{p}), \ldots, \lambda_K(\boldsymbol{p})]^T$ be the vector of the optimal scalar coefficients of the OFMs, as given in (S4). Let $\boldsymbol{D}(\boldsymbol{p})$ be the matrix of cost vectors, as in the proof of Theorem 1. Now, we may rewrite (1) as the LP of maximising the sum of the OFM usage, subject to the enzymatic constraints being active, and non-negativity of the OFM usage:

$$\begin{aligned} \underset{j}{\text{maximise}} \quad & \sum_{j=1}^{r} \lambda_j(\boldsymbol{p}) \\ \text{subject to} \quad & \boldsymbol{D}(\boldsymbol{p})\boldsymbol{\lambda}(\boldsymbol{p}) = \mathbf{1} \\ & \lambda_j(\boldsymbol{p}) \geq 0 \;\; \forall j \end{aligned} \tag{S9}$$

Since the fluxes in a pruned model are necessarily all positive, we do not in fact need the inequality constraint on $\boldsymbol{\lambda}$, and can thus use Lagrange multipliers to calculate the optimum.

We define the objective function f and the constraints function $\boldsymbol{g}$ as

$$\begin{aligned} f(\boldsymbol{\lambda}) &:= \sum_{j=1}^{r} \lambda_j(\boldsymbol{p}) \\ \boldsymbol{g}(\boldsymbol{\lambda}) &:= \boldsymbol{D}(\boldsymbol{p})\boldsymbol{\lambda}(\boldsymbol{p}) - \mathbf{1} \end{aligned} \tag{S10}$$

Write the Lagrangian $\mathcal{L}$, where $\boldsymbol{\nu}$ is the Lagrange multiplier:

$$\begin{aligned} \mathcal{L}(\boldsymbol{\lambda}, \boldsymbol{\nu}) &= f(\boldsymbol{\lambda}) + \boldsymbol{g}(\boldsymbol{\lambda})^T \boldsymbol{\nu} \\ &= \sum_{j=1}^{r} \lambda_j(\boldsymbol{p}) + \; (\boldsymbol{D}(\boldsymbol{p})\boldsymbol{\lambda}(\boldsymbol{p}) - \mathbf{1})^T \boldsymbol{\nu}(\boldsymbol{p}) \end{aligned} \tag{S11}$$

Using the Lagrange multiplier method, we require the following two conditions for optimality of $\boldsymbol{\lambda}$:

$$\begin{aligned} \nabla_{\boldsymbol{\lambda}}\mathcal{L} &= \quad \mathbf{1} + \boldsymbol{D}(\boldsymbol{p})^T\boldsymbol{\nu} = \mathbf{0} \\ \nabla_{\boldsymbol{\nu}}\mathcal{L} &= \boldsymbol{D}(\boldsymbol{p})\boldsymbol{\lambda}(\boldsymbol{p}) - \mathbf{1} = \mathbf{0} \end{aligned} \tag{S12}$$

We can incorporate the Lagrange optimality conditions (S12) into a new function $\boldsymbol{L}$, which is constructed to obey the same conditions:

$$\boldsymbol{L}(\boldsymbol{\lambda}(\boldsymbol{p}), \boldsymbol{p}, \boldsymbol{\nu}) = \begin{bmatrix} \mathbf{1} + \boldsymbol{D}(\boldsymbol{p})^T\boldsymbol{\nu} \\ \boldsymbol{D}(\boldsymbol{p})\boldsymbol{\lambda}(\boldsymbol{p}) - \mathbf{1} \end{bmatrix} = \begin{bmatrix} \mathbf{0} \\ \mathbf{0} \end{bmatrix} \tag{S13}$$

We seek to find the derivatives of the optimal $\boldsymbol{\lambda}$ with respect to $\boldsymbol{p}$, so we define $\boldsymbol{z} = (\boldsymbol{\lambda}, \boldsymbol{\nu})$ and implicitly differentiate $\boldsymbol{L}$ at the optimum $\boldsymbol{z}^*$ to yield:

$$\begin{aligned} \frac{\partial \boldsymbol{L}}{\partial \boldsymbol{z}^*}\frac{d\boldsymbol{z}^*}{d\boldsymbol{p}} + \frac{\partial \boldsymbol{L}}{\partial \boldsymbol{p}} &= \mathbf{0} \\ \frac{d\boldsymbol{z}^*}{d\boldsymbol{p}} &= -\left(\frac{\partial \boldsymbol{L}}{\partial \boldsymbol{z}^*}\right)^{-1}\frac{\partial \boldsymbol{L}}{\partial \boldsymbol{p}} \end{aligned} \tag{S14}$$

We can calculate the value of $\frac{\partial \boldsymbol{L}}{\partial \boldsymbol{z}^*}$, and we can use automatic or symbolic differentiation to efficiently calculate $\frac{\partial \boldsymbol{L}}{\partial \boldsymbol{p}}$ Moses and Churavy (2020); Revels et al. (2016). We may now extract the values of $\frac{d\boldsymbol{\lambda}^*}{d\boldsymbol{p}}$ from $\frac{d\boldsymbol{z}^*}{d\boldsymbol{p}}$, by taking the columns corresponding to the indices in $\boldsymbol{\lambda}$, since $\boldsymbol{z} = (\boldsymbol{\lambda}, \boldsymbol{\nu})$. The values of $\frac{d\boldsymbol{\lambda}^*}{d\boldsymbol{p}}$ are precisely the unscaled sensitivities of OFM usage to model parameters $\boldsymbol{p}$.

S2.4 Metabolic control analysis

A major difference between our approach and that of classical metabolic control analysis of ODE models is that the enzyme constraints cause implicit dependencies between fluxes and turnover numbers of different reactions.

In MCA theory Kacser and Burns (1973), it is assumed that reaction i is only directly affected by parameter i, that is,

$$\frac{\partial v_i}{\partial p_i} \neq 0, \quad \frac{\partial v_i}{\partial p_j} = 0 \ \text{ for any } \ i \neq j. \tag{S15}$$

In our model setup (3) this is not the case. A change in the kinetic parameter p_j affects the concentration of enzyme e_j, which in turn can indirectly affect the optimal concentration of every other enzyme in the solution, due to the total enzyme constraints. Therefore, the summation theorem from MCA for ODE models Heinrich and Rapoport (1974), which states that normalised flux control coefficients sum to 1, will not necessarily hold.

S3 Non-unique optimal solutions

Our method takes an optimal solution to an enzyme-constrained genome-scale metabolic model and prunes inactive reactions and enzymes to ensure that this is a unique optimal solution. Before pruning, it is in general not guaranteed that there is a unique optimal solution. Choosing which optimal solution to analyse is vital in any analysis of constraint-based models. Here we wish to discuss this in the context of sensitivity analysis.

The issue of which optimal solution sensitivities should be calculated for is a consideration in any sensitivity analysis of an optimisation problem with a non-unique optimal solution. Should

a parameter change cause a switch to a different optimal solution, there will be discontinuity or smoothness challenges. In any method, the quantity $\frac{\partial v}{\partial p}$ will be either undefined, or difficult to interpret (a generalised derivative), when the function $v(p)$ is discontinuous or non-smooth at p. When we prune the model, we make the assumption that the set of reactions used in an optimal solution is stable to infinitesimal parameter changes. We only allow one optimal solution, forcing $v(p)$ to be smooth and differentiable. We can therefore calculate the sensitivity of the variables in the chosen solution to the model parameters. These sensitivities are interpretable within our assumption. In frameworks without pruning, such as finite differentiation or shadow prices, the switching between optimal solutions can occur. This renders sensitivities very difficult to interpret. Investigation into switching points of metabolism is beyond the scope of this paper, but would be valuable in furthering understanding of whole cell metabolism.

In the case of classic FBA models, running flux variability analysis (FVA) often reveals highly variable optimal fluxes. This reveals that optimal solutions can vary greatly from one another. However, this does not appear to be as significant an issue with enzyme-constrained models. When running FVA on ecFBA models, we find that the variability of optimal fluxes is greatly reduced. This is due to the different costs associated with different enzymes. Using realistic k_{cat}-values and gene product molar masses causes the optimal solution space of ecFBA models to be vastly reduced compared to FBA models.

If an ecFBA model has multiple optimal solutions, deciding which optimal solution to differentiate is at the discretion of the reader. It may be of interest to take a sample of optimal solutions. For example, by constraining one or more fluxes to their upper or lower optimal flux values and solving the new LP, one would find new optimal solutions to the original LP. These alternative optimal solutions could all be pruned and differentiated, so that the sensitivities of both OFM usage and reaction fluxes in these different optimal solutions could be compared to find some sense of an 'average' sensitivity across solutions.

References

A. Agrawal, S. Barratt, S. Boyd, E. Busseti, and W. M. Moursi. Differentiating through a cone program. *arXiv preprint arXiv:1904.09043*, 2019.

D. H. De Groot, C. Van Boxtel, R. Planqué, F. J. Bruggeman, and B. Teusink. The number of active metabolic pathways is bounded by the number of cellular constraints at maximal metabolic rates. *PLOS Computational Biology*, 15(3):e1006858, 2019.

J. Gagneur and S. Klamt. Computation of elementary modes: a unifying framework and the new binary approach. *BMC bioinformatics*, 5(1):1–21, 2004.

R. Heinrich and T. A. Rapoport. A linear steady-state treatment of enzymatic chains: general properties, control and effector strength. *European journal of biochemistry*, 42(1):89–95, 1974.

H. Kacser and J. Burns. Rate control of biological processes. In *Symp. Soc. Exp. Biol*, volume 27, pages 65–104, 1973.

W. Moses and V. Churavy. Instead of rewriting foreign code for machine learning, automatically synthesize fast gradients. *Advances in neural information processing systems*, 33:12472–12485, 2020.

J. Revels, M. Lubin, and T. Papamarkou. Forward-mode automatic differentiation in julia. *arXiv preprint arXiv:1607.07892*, 2016.

S. E. Wilken, M. Besancon, M. Kratochvíl, C. A. F. Kuate, C. Trefois, W. Gu, O. Ebenhöh, et al. Interrogating the effect of enzyme kinetics on metabolism using differentiable constraint-based models. *Metabolic engineering*, 74:72–82, 2022.
